# The power and the promise of organoid models for cancer precision medicine with next-generation functional diagnostics and pharmaceutical exploitation

**DOI:** 10.1016/j.tranon.2021.101126

**Published:** 2021-05-18

**Authors:** Yu-Shui Ma, Xiao-Li Yang, Rui Xin, Ting-Miao Wu, Yi Shi, Dan Dan Zhang, Hui-Min Wang, Pei-Yao Wang, Ji-Bin Liu, Da Fu

**Affiliations:** a National Engineering Laboratory for Deep Process of Rice and Byproducts, College of Food Science and Engineering, Central South University of Forestry and Technology, Changsha 410004, Hunan, China; b Cancer Institute, Nantong Tumor Hospital, Affiliated Tumor Hospital of Nantong University, Nantong 226631, China; c International Cooperation Laboratory on Signal Transduction, Eastern Hepatobiliary Surgery Hospital/Institute, National Center for Liver Cancer, the Second Military Medical University, Shanghai 200433, China; d Central Laboratory for Medical Research, Shanghai Tenth People's Hospital, Tongji University School of Medicine, Shanghai 200072, China; e Department of Radiology, The Forth Affiliated Hospital of Anhui Medical University, Hefei 230012, China

**Keywords:** Organoid, *In vitro model*, Cancer, Drug screening, Precision medicine

## Abstract

As organ-specific three-dimensional cell clusters derived from cancer tissue or cancer-specific stem cells, cancer-derived organoids are organized in the same manner of the cell sorting and spatial lineage restriction *in vivo*, making them ideal for simulating the characteristics of cancer and the heterogeneity of cancer cells *in vivo*. Besides the applications as a new *in vitro* model to study the physiological characteristics of normal tissues and organs, organoids are also used for *in vivo* cancer cell characterization, anti-cancer drug screening, and precision medicine. However, organoid cultures are not without limitations, *i.e.*, the lack of nerves, blood vessels, and immune cells. As a result, organoids could not fully replicate the characteristics of organs but partially simulate the disease process. This review attempts to provide insights into the organoid models for cancer precision medicine.

## Graphical abstract

**Figure:**
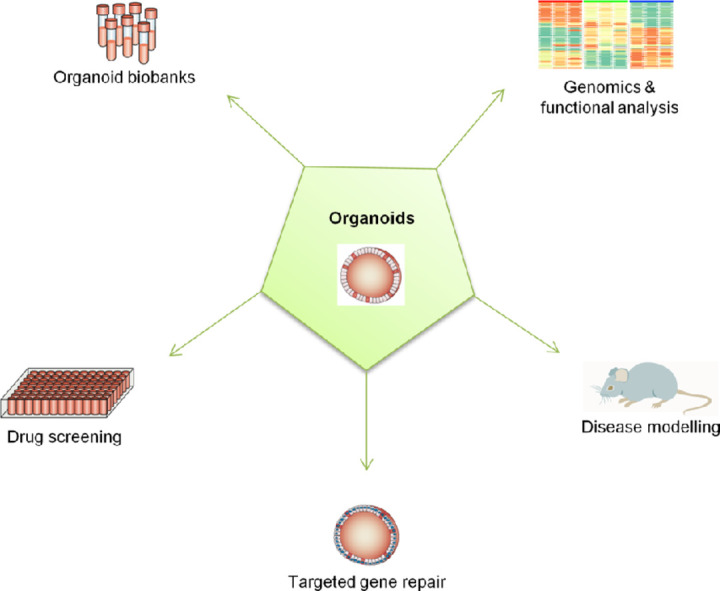

## Background

**Fig. 1:**
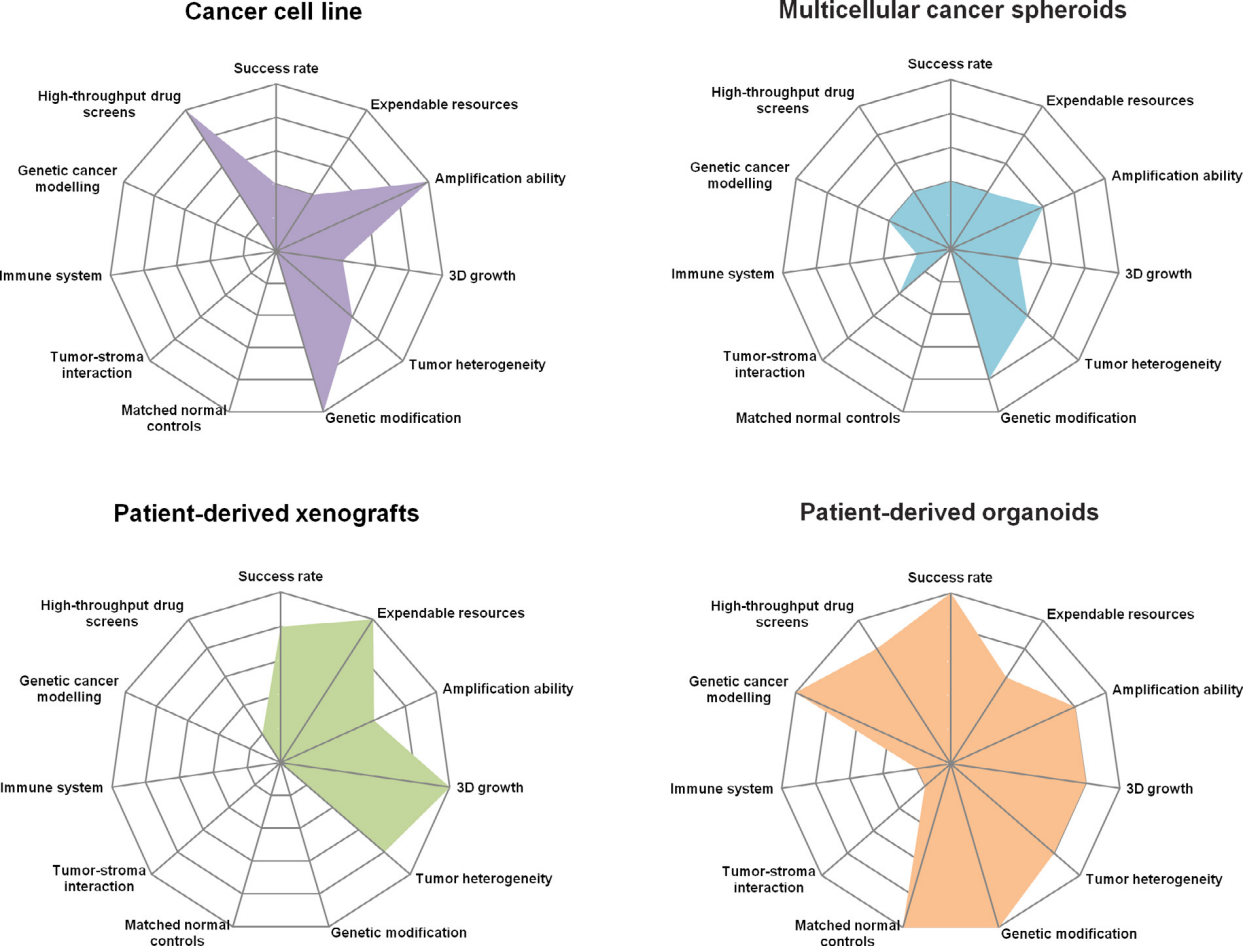
Characteristics and multi-dimensional comparison of the commonly used models in cancer research. Characteristics and multi-dimensional comparison of patient-derived cancer cell lines, multicellular cancer spheroids, patient-derived xenografts, and cancer patient-derived organoids models. Although these models have their advantages and disadvantages, they are all of great significance to cancer research.

Although a global health threat, research on the pathogenesis and treatment of cancer is still ongoing, in which two-dimensional (2D) cell culture and xenotransplantation of cancer cells provide the materials for experiments [1], [2], [3]. However, due to the limitations of the existing technologies, the requirements of more in-depth experimental research are still not met [4]. A high-quality and high-efficiency culture scheme is urgently needed to provide more representative experimental materials for research. Establishing predictive pre-clinical models would allow for more accurate and practical therapeutic drug development. Pharmacological development and advancing personalized medicine using patient-derived xenografts (PDXs) relies on producing mouse models, which are extensively used as *in vivo* system for biomedical research. However, due to the significant differences between rodents and human, it is impossible to translate most of the findings from mouse models to human. The patient cancer-derived organoids (PCDOs) could be a possible answer to that need [5]. Organoids are expected to play an important role in cancer precision medicine, new drug development, and the study of cancer pathogenesis due to their high flux, low cost, and genetic stability [6], [7], [8], [9] (Fig. 1).

**Fig. 2:**
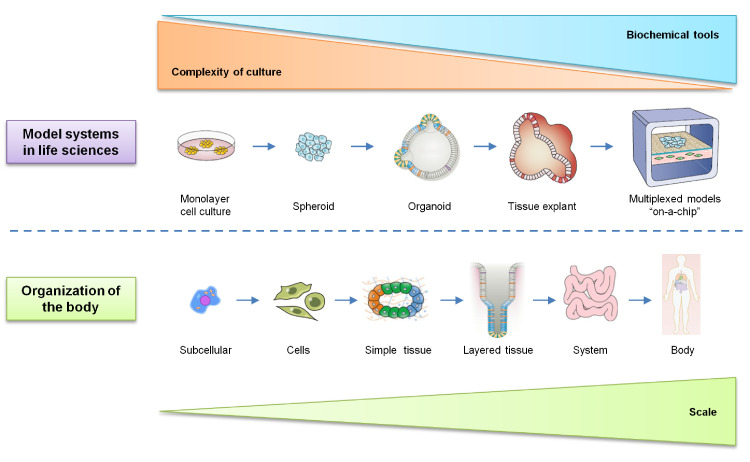
Model systems in life sciences. Organisms comprise a hierarchy of systems from the subcellular level to the whole body. Many models have been developed across this organismal hierarchy in life sciences to address specific questions across biology and medicine. Each model system possesses unique attributes. In general, with increasing scale comes the increasing system complexity and challenges in cell culture and the reduced availability of biochemical and quantitative tools.

PCDOs are small *in vitro* tissue or organ analogs derived from patient cancer tissue or cancer-specific stem cells cultured using the self-renewal and differentiation ability of stem cells [10]. To date, a variety of PCDOs have been successfully cultivated under three-dimensional (3D) culture conditions, including colon cancer organoids [11], prostate cancer organoids [12], gastric cancer (GC) organoids [13], breast cancer organoids [14], pancreatic cancer organoids [15], ovarian cancer organoids [16], kidney cancer organoids [17], and bladder cancer organoids [18]. PCDOs have simple culturing procedures, high tumorigenesis rate, and suitability for high-throughput drug screening and genetic operation [19]. PCDOs maintain the original cancer structure and heterogeneity and have been widely used in cancer research [20]. PCDOs provide a unique opportunity to incorporate moderate system complexity while still affording the many tools for probing the structure and function [21]. Compared to tissue explants, organoids mimic the cell-cell and cell-matrix interactions while maintaining the capacity for long-term cultures thanks to the maintained signaling cues for survival [22]. With the application of PCDOs in drug screening, precision medicine has become a reality (Fig. 2). However, organoid cultures are not without limitations, such as the lack of nerves, blood vessels, and immune cells in the model. As a result, the characteristics of organs are not fully depicted, and the disease processes are only partially simulated. This review aims to provide insights into the organoid models for cancer precision medicine.

## Development of organoid technology

**Fig. 3:**
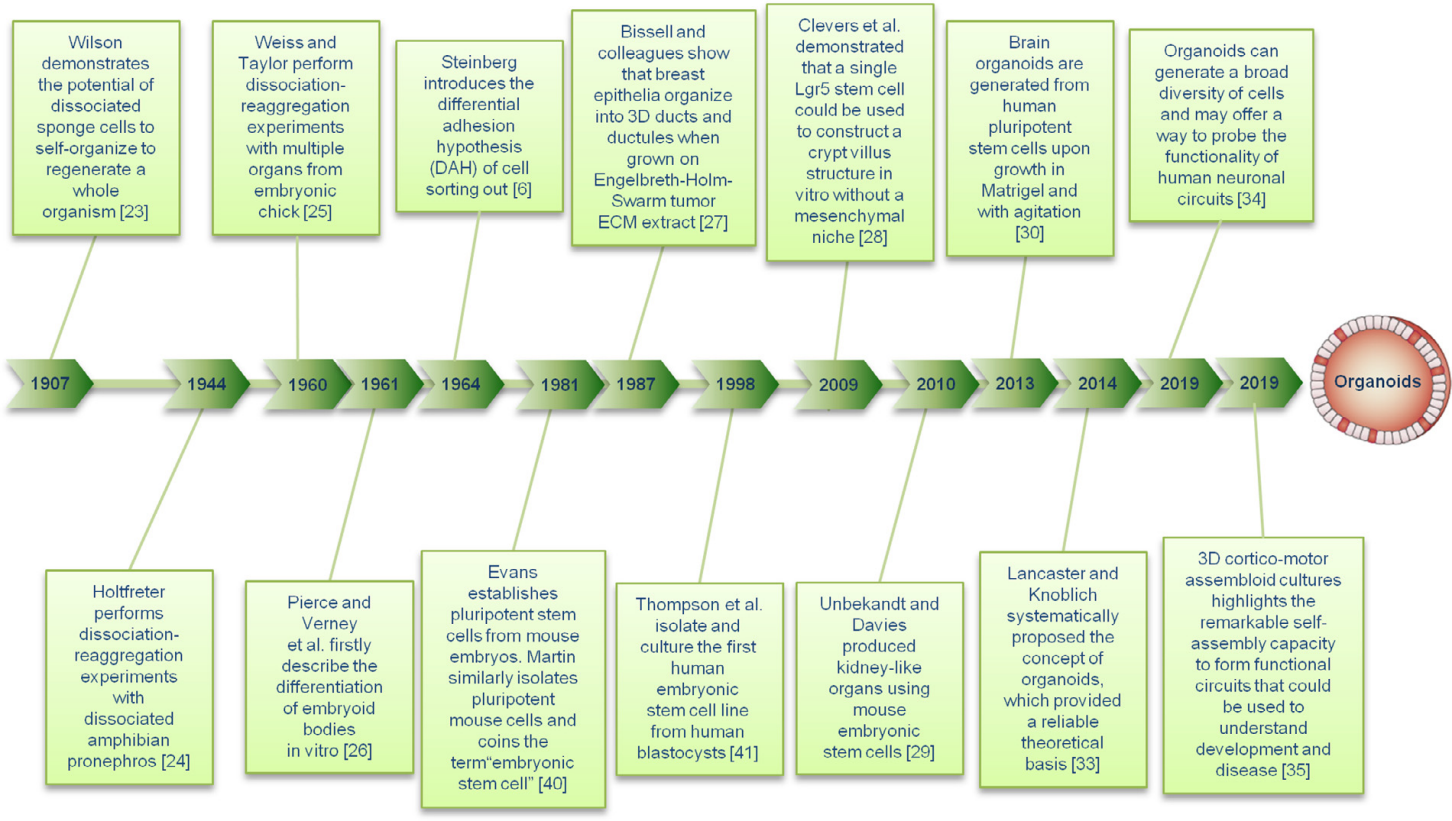
History of organoid methodologies. The key events leading to the various organoid methodologies are listed.

In 1907, Wilson demonstrated for the first time that mechanically isolated sponge cells could regroup and self-organize to form a complete organism [23]. The advent of organoids was marked by the transformation from 2D medium to 3D medium, making the complex 3D organ structure possible [24]. In studies conducted in the early 1960s, dissociated cells from the developing chick kidney were utilized to form reaggregates that recapitulate the virtually complete renal development [25,26]. Since 1987, researchers have used different stem cells to produce a large number of cells similar to normal tissues and organs in the body [27]. In 2009, Clevers et al. demonstrated that a single Lgr5 stem cell could be used to construct a crypt-villus structure *in vitro* without a mesenchymal niche [28]. In 2010, Unbekandt and Davies produced kidney-like organs using mouse embryonic stem cells [29]. In 2013, Madeline Lancaster of the Austrian Academy of Sciences demonstrated that brain tissue organoids could be produced by culturing the human pluripotent stem cells in Matrigel [30]. In 2014, Shkumatov et al. showed that physiological stiffness promotes the 3D formation of embryonic stem cells and cardiomyocyte differentiation [31]. Takebe et al. found a general method for the formation of organ buds from different tissues by combining tissue-specific progenitor cells or endothelial cell-related tissue samples and mesenchymal stem cells derived from pluripotent stem cells [32]. They believed that the immature tissues or organ buds produced by the self-organization condensation principle might be an effective method for the functional reconstruction of mature organs compared with the coagulants produced by cells at higher stages. Although attempts have been made to describe the organogenesis process through developmental biology experiments, it remained unclear until 2014 when Lancaster and Knoblich systematically proposed the concept of organoids [33] (Fig. 3). This concept has provided a reliable theoretical basis for the development of biomedicine and the understanding of diseases, especially cancer [34,35].

### Culture and cell source of organoids

**Fig. 4:**
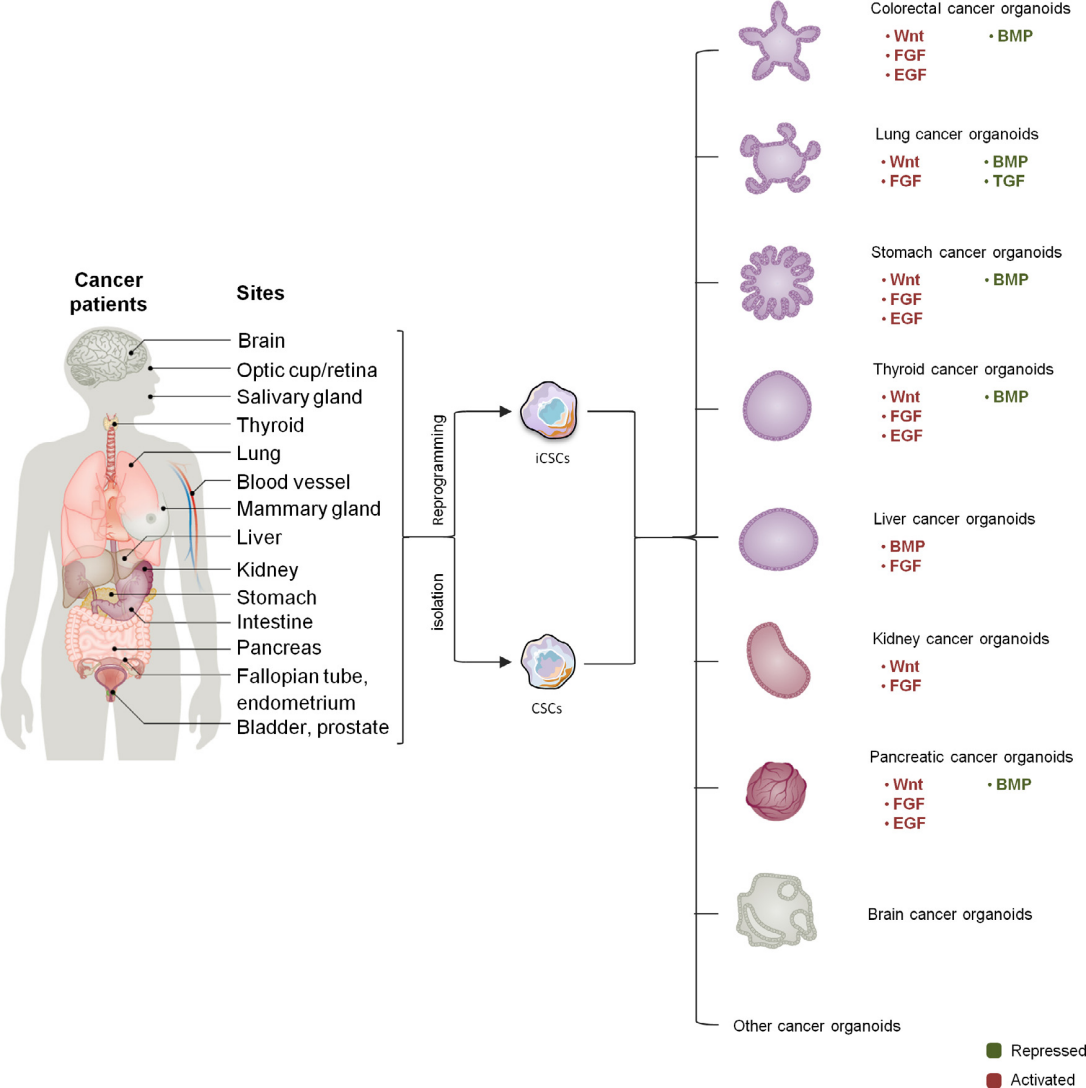
The key growth factors for PCDOs establishment. PCDOs are established following the directed differentiation of cancer cells with stem cell characteristics, which requires germ-layer specification, induction, and maturation. Specific growth and signaling factors are adopted to obtain the specific cell types that form the desired organ.

The commonly used organoid culture system is matrix glue. The basement membrane matrix is extracted from mouse sarcoma rich in extracellular matrix protein [36]. The main components include laminin, type IV collagen, nestin, heparin sulfate glycoprotein, growth factor, and matrix metalloproteinase [37]. At room temperature, Matrigel polymerizes to form a 3D matrix with biological activity, which simulates the structure, composition, physical properties, and functions of the cell basement membrane *in vivo* and is conducive to cell culture and differentiation *in vitro* [38]*.* Epidermal growth factor, basic fibroblast growth factor, insulin and transferrin, and dexamethasone and Wnt activator regulate the formation of organoids and cell differentiation [39] (Fig. 4).

Organoids could derive from one or more stem cells, such as embryonic stem cells (ESCs), induced pluripotent stem cells (iPSCs), and adult stem cells (ASCs) [40], [41], [42]. As the biological characteristics of different stem cells are different, the application prospects of different organoids are also different. A suitable 3D culture system could direct the differentiation of ESCs into embryoid bodies. Then relevant signal factors could be added according to different organ development purposes to induce cell self-organization and position rearrangement to develop organoid bodies [43]. Organoids could simulate organ development *in vivo* and help to study the morphological characteristics and organ transplantation [44].

### Biological characteristics of organoids

In 2009, the first PCDOs model was established with small intestinal crypt stem cells [28]. Subsequently, models of breast, prostate, liver, kidney, and lung cancers were established [45], [46], [47], [48], [49]. Organoids have the following biological characteristics. (1) Cell proliferation, differentiation, and self-renewal: stem cells are the basis for establishing PCDOs, which proliferate and differentiate into multi-functional cells under specific organ development conditions to realize self-renewal within tissues [50]. (2) Cell self-assembly: after differentiation, stem cells migrate to specific locations and arrange in an orderly manner under the regulation of signal factors to form tissues similar to organ structure [51]. (3) Physiological activity of organs: after organ structure formation, the corresponding biological signals are established to reproduce multicellular physiological activities and signal regulation in organs to a certain extent. Cancer organoids have been shown to retain the key genetic and phenotypic characteristics of the original tissue and the tumor subtype and maintain the intratumoral heterogeneity; therefore, cancer organoids have the potential to be used as predictors for precision medicine response [52]. (4) Long-term culture and genetic stability: During long-term culture, the stem cells maintain cell renewal and genetic stability [53]. (5) Genetic heterogeneity: Tumor-derived organoids maintain the genetic heterogeneity of the primary tumor tissue over time; thus, the predictive value of therapeutic testing for individual patients is auspicious. The first organoid bank for pediatric kidney cancer contains the tumors and the corresponding organoid cultures from more than 50 children with Wilms and non-Wilms tumors. The extensive characterization of this biobank revealed that the organoids recapitulated patient copy number alterations and mutational signatures and that the patient-specific drug sensitivities were retained [54]. The organoids retained the genetic and phenotypic features of the original tumor and thus superior to the corresponding spheroid culture as only the organoids grown in cell suspension embedded in Matrigel could reproduce the tumor morphology and architecture. Therefore, this new technology has simple operation and high throughput compared to traditional 2D cell culture, making it a good representation of the parental tumors.

### Establishment of PCDOs

PCDOs could retain the biological characteristics and heterogeneity of cancer tissue and have a stable genome and short culture cycles after multiple passages, making it an ideal model for cancer research [55], [56], [57], [58]. At present, cancer cells are mainly used to cultivate PCDOs. In 2015, Boj et al. established the pancreatic PCDOs [59]. After targeted sequencing of more than 2000 cancer-related genes, it was found that PCDOs have cancer-related gene mutations that could maintain the physiological and structural characteristics of cancers. In 2017, Broutier et al. [60] cultivated organoids with three different subtypes of primary liver cancer cells and found that PCDOs retained the histological structure and genomic composition of the original cancer cells and that the different cancer tissues and subtypes were distinguishable even after long-term culture. After transplanting into mice, PCDOs retained the histological structure and expression profile of cancer and showed their potential to metastasize *in vivo*. PCDOs could simulate the pathological characteristics, development process, and biological signals of cancers [61], [62], [63]. Genetic engineering technology was used to study cancer organs and defined the interaction among cancer cells, healthy cells, and matrix, which could benefit the development of precision medicine and anti-cancer drug screening [64], [65], [66].

### PCDOs for simulating the tumor microenvironment

The tumor microenvironment (TME) comprises cancer cells, stromal cells, immune cells, and endothelial cells [67], [68], [69]. TME participates in the whole process of cancer occurrence, development, and drug reaction [70]. Although the difficulties above could be overcome with cancer animal models, the relevant microenvironment factors are hard to manipulate, and real-time dynamic high-resolution observation is hard to achieve. In recent studies, researchers co-cultured PCDOs with other types of cells in TME, such as fibroblasts and immune cells, to characterize the TME *in vitro* [71], [72], [73]. The interaction between pathogen clearance-defective receptor-1 and programmed cell death protein 1 can be studied by co-culturing the GC tissues and the innate immune cells derived from the transgenic mice model and simulate the GC TME [74]. Chen et al. [75] cultured the stomach-like organs of p53-deficient mice containing epithelial cells and the mesenchymal matrix to simulate the TME and the signal transduction. The transcriptome of 4391 cells was determined using the single-cell RNA sequencing technology; the presence of epithelial cells, fibroblasts, and phagocytes were identified in PCDOs; the system was found suitable for studying the TME and the immune response of gastric tissue [76]. Esser et al. cultured the clear cell renal cell carcinoma patient-derived organoids in an air-liquid interface system to validate their close similarity to the corresponding tumor and found that immune cells and stromal cells within the microenvironment could be identified [77].

### PCDOs for the study of tumorigenesis and clonal evolution

Tumorigenesis is the result of gene mutation accumulation [78,79]. The PCDOs model could help understanding how mutations occur and accumulate in the process of cancer development. Organoids could be cultivated from normal tissue and cancer tissue at the same time. The former has relatively stable genetic information and could be a good control model for studying the origin of cancer mutation signal.

Besides the application of examining normal development, organoids have also been used to study tumorigenesis. In most studies on cancer that adopt organoids, primary carcinoma samples have been generated under ASC-organoid conditions. However, CRISPR mutagenesis technology has been applied to PSC-based organoids to generate cancer-causing mutations, for example, to model human brain tumors. In addition, Fine and colleagues have explored PSC-derived mini-brains as an environment for growing patient-derived glioblastoma cells [80].

Jager et al. [81] used single stem cells to cultivate the corresponding organs and explore the gene mutations in the development of the small intestine, colorectal, and liver. Whole-genome sequencing analysis of different-aged organs revealed that the mutation rate of different stem cells was the same; the mutation types of the small intestine and colorectal stem cells differed from those of the liver as the continuous renewal of small intestine and colorectal stem cells leads to gene-specific deamination mutation. This gene mutation is also one of the main reasons for colorectal cancer [82]. Therefore, the application of PCDOs provides a novel basis for the clinical exploration of different mutation treatment methods [83], [84], [85].

### PCDOs for drug screening and development

Cancer is a highly heterogeneous disease [86]. Therefore, the physiological, pathological, and clinical drug response models of cancer patients must be established to evaluate the potential drug effects and the effectiveness of the treatment. As PCDOs retain the heterogeneity and histological characteristics of the original cancers, it has become an ideal model for testing new anti-cancer drugs [87].

The cost of isolating and culturing the cancer patient organ samples and constructing the PCDO biological sample bank is relatively low; the time spent is short; thus, PCDOs are more suitable for gene operation than the cancer xenotransplantation model [88]. PCDOs could be used for large-scale high-throughput drug screening and development. With close morphological/genetic resemblance to the progenitor tissue and the long-term stability in culture, PCDOs are increasingly employed as the pre-clinical model for drug screening and radiosensitivity assays. ERK inhibitor, or HER inhibitor combined with a MEK inhibitor, was used respectively in colorectal cancer-derived organs with RAS mutation [89]. The results showed that the three-drug regimens could effectively inhibit cancer growth. However, studies have found that these treatments only lead to cell cycle arrest rather than apoptosis of PCDO cells. Therefore, when the drug is stopped, cancer cells proliferate. When combined with EGFR pathway inhibitors, RAS-mutated colorectal cancer cells can be sensitized again, which provides a new choice for the clinical treatment of colorectal cancer patients.

Organoids could derive from both cancer tissues and normal adult tissues [90]. Therefore, when applied to drug development, organoids may help to screen drugs specific to cancer cells without damaging normal cells. Drug-induced hepatotoxicity is generally mediated by cytochrome P450 enzyme, and liver-derived organoids could express cytochrome P450 enzyme close to the physiological level in the process of induced differentiation [91]. Therefore, liver-derived organoids may be adopted to test drug hepatotoxicity in pre-clinical trials. Similarly, iPSC-derived cardiac organoids could be used to test the cardiotoxicity of drugs, and iPSC-derived kidney organoids could be used in renal toxicology research.

### PCDOs for cancer precision medicine

Currently, therapeutic drugs for different stages of cancer are being developed rapidly. However, prescribed therapy is usually based on the general success rate of the drug rather than the response of specific patients to the drug. Moreover, drug testing with conventional monoculture pre-clinical models is misleading and most likely responsible for the high failure rate of phase 3 trials. It is, therefore, a clinical and research priority to construct a reliable model that can predict patient responsiveness and enable patient-tailored treatment strategies. Although the development of disease staging, pathological typing, gene sequencing, and molecular typing is helpful to guide the clinical treatment of cancer patients, effective tools are still needed to support the prediction of drug response of specific individuals [92]. Cultivating cancer organoids could help directly research the cancer samples from patients with good passage stability. Moreover, the organoids cultured from patient tissues could preserve the tissue structure, gene expression, and genome panorama of the original cancers. Even in the same medium conditions for long-term expansion, the organoids still retain the characteristics of different cancer tissues and subtypes.

**Fig. 5:**
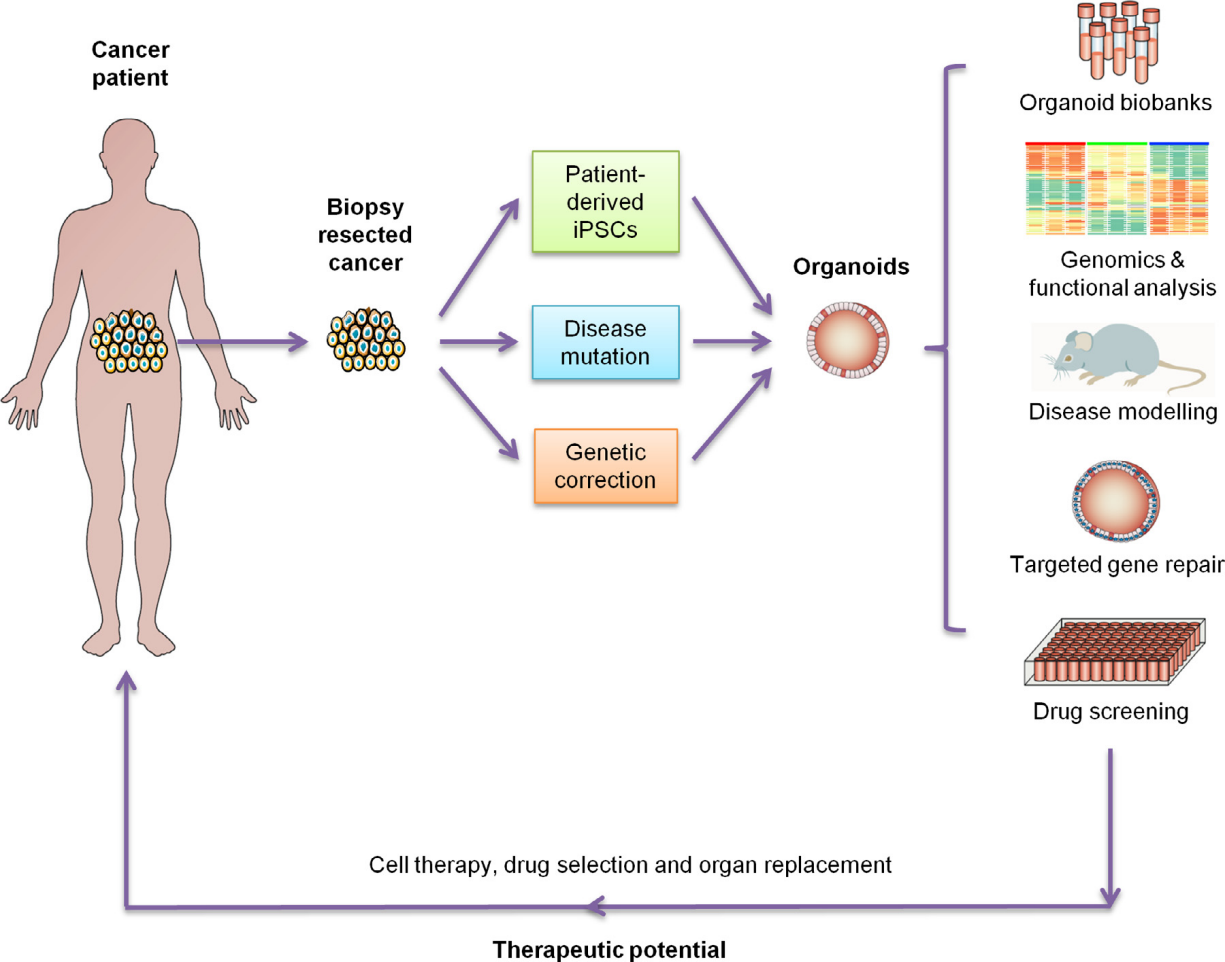
Applications of organoid culture. The organoid culture enables specific fundamental and clinical applications. The organoids derived from cancer tissue mimic the tissue organization and turnover in cancer and therefore enables the studies on tissue pathophysiology and functional assays. Also, organoids can be expanded and used to test drug efficacy and toxicity or personalized medicine. Expanded organoids from a single patient might also be used for bioengineering and cell therapy, possibly in combination with targeted gene repair.

Compared with traditional precision medicine, such as gene expression detection and drug sensitivity test, the application time of cancer organoids is significantly shorter, facilitating the real-time evaluation of the effect on each patient [93]. The relevant experimental data based on this model could be quickly transformed into the basis of clinical decision-making, which not only provides evidence for the optimal treatment combination but also reduces ineffective treatment (Fig. 5).

Seidlitz et al. [94] used a tamoxifen-induced CreERT2 system to construct different subtypes of GC transgenic mice. Cultivation of the organoids from different subtypes of GC mice revealed that the organoids derived from gastric cancer mice were resistant to docetaxel, whereas those from chromosome unstable GC were more resistant to trametinib. Steele et al. [95] established an organoid sample library based on the samples from seven patients with GC. RNA sequencing showed that the transcriptome information of organ-like tissues from GC patients was similar to that of the original cancer tissues of the patients. The standard chemotherapeutic drugs (epirubicin, oxaliplatin, and 5-fluorouracil) were used to treat the constructed GC-like organs, and the sensitivity of the patients to the several chemotherapeutic drugs was compared. The established GC-like organs were helpful to predict patient sensitivity to chemotherapy drugs. Thus, PCDOs better recapitulate native tumors and may be superior models to identify and test novel anti-cancer drugs. The development of high-throughput drug screening methods in PCDOs is just beginning to be explored.

### Outlook

In cancer research, PCDOs could effectively simulate the dynamic pathological changes of cancer cells *in vivo*. The interaction between host and pathogen could be simulated by the microinjection of pathogenic microorganisms into the epithelial cavity of the organoids. Co-culture of PCDOs with host immune cells and other cells can provide convenient conditions for revealing the role of the TME in tumorigenesis and development. Compared with traditional cancer cell modeling methods, PCDOs could maintain genomic stability and cancer heterogeneity, which provides a model closer to the characteristics of the original cancer tissue.

The emergence of patient-derived xenograft (PDX) models as a surrogate, translational, and functional representation of the patient with cancer has led to the advances in drug discovery and testing of novel targeted approaches and combination therapies. However, current established PDX models fail to represent the diverse patient population and, most importantly, the specific ethnic patient populations that have higher rates of incidence and mortality. When compared with the cancer xenotransplantation model, cancer organoids have a lower cost and are less time-consuming. In addition, establishing a PCDO biological sample bank could provide an optimization model for clinical drug high-throughput screening and precision medicine and contribute to research cooperation among different countries, research institutions, and teams.

Although PCDOs are unique and advantageous *in vitro* models, there are still limitations and shortcomings. The establishment of PCDOs requires a variety of technical and logistical support. The technical requirements for establishing a PCDO sample library by separating the cancer tissue of patients are high; obtaining enough cancer samples is difficult due to the limited number of patients and the fact that samples cannot be obtained by surgery from patients with advanced cancer. In TME, there are cancer tissue and many supporting cells, stroma, and neovascularization, which are involved in cancer growth and metastasis. PCDOs were cultivated in the best growth environment and growth-promoting matrix and lack angiogenesis, so they can not completely simulate the TME of cancer tissue *in vivo*. Further technical optimization is needed based on PCDOs culture in future studies to provide the best model for revealing the occurrence and development mechanism of cancer, thereby leading to the development in cancer prevention, treatment drugs, and precision medicine.

## Conclusions

PCDOs recapitulate the spatial arrangement of the original tissue and simulate the characteristics of cancer and the heterogeneity of cancer cells *in vivo*. Up to now, researchers have successfully produced a variety of PCDOs, including colon cancer, prostate cancer, gastric cancer, breast cancer, pancreatic cancer, ovarian cancer, kidney cancer, and bladder cancer. As a new type of *in vitro* model, organoids maintain the characteristics of cancer cells *in vivo* and could be used to study the physiological characteristics of normal tissues and organs. PCDOs could also be used for anti-cancer drug screening and precision medicine.

## Declaration of Competing Interest

The authors declare that they have no competing interests.
